# Optimization of Growth and Carotenoid Production by *Haloferax mediterranei* Using Response Surface Methodology

**DOI:** 10.3390/md16100372

**Published:** 2018-10-09

**Authors:** Zaida Montero-Lobato, Adrián Ramos-Merchante, Juan Luis Fuentes, Ana Sayago, Ángeles Fernández-Recamales, Rosa María Martínez-Espinosa, José María Vega, Carlos Vílchez, Inés Garbayo

**Affiliations:** 1 Algal Biotechnology Group, CIDERTA and RENSMA, University of Huelva, 21071 Huelva, Spain; zaida.montero@dqcm.uhu.es (Z.M.-L.); jlfuentes@dqcm.uhu.es (J.L.F.); garbayo@dqcm.uhu.es (I.G.); 2 Department of Integrated Sciences, Faculty of Experimental Sciences, University of Huelva, 21007 Huelva, Spain; adrian.ramos@ciecema.uhu.es; 3 Department of Chemistry, Faculty of Experimental Sciences, University of Huelva, 21007 Huelva, Spain; ana.sayago@dqcm.uhu.es (A.S.); recamale@dqcm.uhu.es (A.F.-R.); 4 Biochemistry and Molecular Biology Division, Agrochemistry and Biochemistry Department, Faculty of Sciences, University of Alicante, E-03080 Alicante, Spain; rosa.martinez@ua.es; 5 Department of Plant Biochemistry and Molecular Biology, Faculty of Chemistry, University of Seville, 41012 Seville, Spain; jmvega@us.es

**Keywords:** bacterioruberin, *Haloferax mediterranei*, response surface methodology (RSM), central composite design (CCD)

## Abstract

*Haloferax mediterranei* produces C50 carotenoids that have strong antioxidant properties. The response surface methodology (RSM) tool helps to accurately analyze the most suitable conditions to maximize C50 carotenoids production by haloarchaea. The effects of temperature (15–50 °C), pH (4−10), and salinity (5–28% NaCl (*w*/*v*)) on the growth and carotenoid content of *H. mediterranei* were analyzed using the RSM approach. Growth was determined by measuring the turbidity at 600 nm. To determine the carotenoid content, harvested cells were lysed by freeze/thawing, then re-suspended in acetone and the total carotenoid content determined by measuring the absorbance at 494 nm. The analysis of carotenoids was performed by an HPLC system coupled with mass spectrometry. The results indicated the theoretical optimal conditions of 36.51 or 36.81 °C, pH of 8.20 or 8.96, and 15.01% or 12.03% (*w*/*v*) salinity for the growth of haloarchaea (OD600 = 12.5 ± 0.64) and production of total carotenoids (3.34 ± 0.29 mg/L), respectively. These conditions were validated experimentally for growth (OD600 = 13.72 ± 0.98) and carotenoid production (3.74 ± 0.20 mg/L). The carotenoid profile showed four isomers of bacterioruberin (89.13%). Our findings suggest that the RSM approach is highly useful for determining optimal conditions for large-scale production of bacterioruberin by haloarchaea.

## 1. Introduction

Carotenoids (carotenes and xanthophylls) are pigments present in all living organisms; however, they are synthesized only by bacteria, algae, fungi, and plants. They comprise a large family of over 700 naturally-occurring pigments characteristically present in leaves, flowers, and fruits of plants, where they play various roles. In plants and algae, they utilize light energy to support the chlorophyll-dependent photosynthetic electron flow inside the chloroplasts. In addition, carotenoids dissipate excess light energy and owing to their antioxidant activity, protect the photosynthetic machinery against photoinhibition caused by free oxygen radicals [1].

Carotenoids also play an important role in human health by acting as provitamin A, which protects against macular degenerative disease and cancer. These effects, coupled with the fact that humans use carotenoids from their diet, make these pigments highly valuable for foods, pharmaceutics, and cosmetics industries. Although they are usually commercially produced by chemical synthesis, microorganisms can also be important alternative sources of carotenoids and their active isomers. β-Carotene, astaxanthin, lutein, and canthaxanthin are C40 carotenoids, which are highly valuable for biotechnological purposes [2,3].

Halophilic archaea include microorganisms that grow optimally in culture media with high salt concentrations of up to 4 M. The family Haloferacaceae comprise non-photosynthetic and largely aerobic heterotrophs, which produce carotenoids as components of their cytoplasmic membranes, especially under conditions of low salinity in the medium [4]. Apart from carotenoids, haloarchaea also produce high-added-value products of biotechnological interest, such as enzymes capable of being active at high temperature and high ionic strength, polysaccharides, polyalkanoates, and polyhydroxybutyrate [5]. In addition, *Haloferax mediterranei* excretes halocins capable of killing other archaea. Halocin H4 is a protein of mass 34.9 kDa that targets the plasma membrane of microorganisms, effecting change in permeability and causing ionic imbalance [6].

Usually, the C50 carotenoid bacterioruberin and its derivatives monoanhydrobacterioruberin and bisanhydrobacterioruberin are the major carotenoids produced by halophilic archaea. These carotenoids may be found as trans and cis isomers [7]. They improve the rigidity and fluidity of the cell membrane [8], and, owing to their strong antioxidant properties, protect the cells from the harmful effects of radiation energy as well as from osmotic stress produced by low salinity in the medium [9,10]. Several halophilic bacteria also produce other carotenoids such as β-carotene, lycopene, and canthaxanthin [11,12]. C50 carotenoids produced by haloarchaea possess higher antioxidant capacity than C40 carotenoids produced by most photosynthetic organisms, due to the higher number of pairs of conjugated double bonds. C50 carotenoids are therefore interesting in food applications and for the pharmaceutical industry. The relative proportion of bacterioruberin content in cells depends on the strain of haloarchaea and the culture conditions used, particularly temperature, pH, and salinity. Other factors such as the addition of selected organic compounds to the culture medium also influences the carotenoid production of halophilic archaea [12]. The culture conditions should be set beforehand to maximize biomass yield and carotenoid production, thereby improving yield and reducing costs [5].

Studies on the biotechnological use of halophilic archaea are scarce, despite the widespread interest in C50 carotenoids, and *H. mediterranei* can be a good candidate due to its ability of growth in a wide range of temperatures, pH, and salinity. Generally, the approach to standardize and optimize the conditions of growth and carotenoid production simultaneously, particularly at large-scale production, is complicated [7,13]. Statistical experimental methods such as central composite design (CCD) and response surface methodology (RSM) can be used in microbial processes to determine the conditions for optimal productivity [14]. We demonstrate that RSM is useful for optimization of conditions for growth rate and carotenoid production by *H. mediterranei* at the laboratory scale. This approach will be valuable for carotenoid production at the industrial scale.

## 2. Results and Discussion

### 2.1. Effect of Air Volume inside the Culture Flasks and Speed of Agitation on the Growth Rate of H. mediterranei

**Figure 1:**
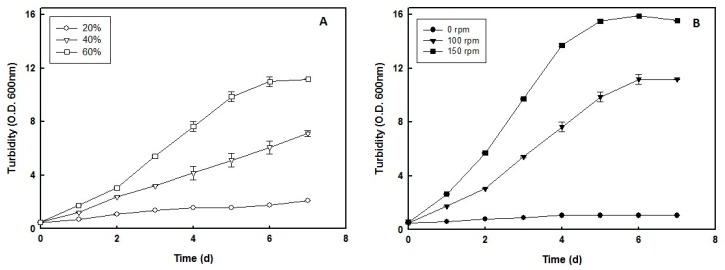
Effect of air phase (**A**) and shaker speed (**B**) of cultures on the growth of *H. mediterranei*. Cells were grown under standard conditions, as stated in Materials and Methods, and using the indicated air phase and shaker speed. When indicated, the turbidity of the culture was determined at 600 nm.

Oxygen supply is essential for optimal growth and carotenoid production in haloarchaea [15]. The influence of air volume inside the culture flasks on the growth rate of *H. mediterranei* was evaluated while all the other culture conditions were kept constant. This study was not aimed at determining the optimal air phase volume for production of *H. mediterranei* as it depends on the cultivation system design and parameters specifically selected in each production process. However, an air phase volume was used in this study so that the oxygen availability allowed *H. mediterranei* to complete growth until the stationary phase. Air occupying 20%, 40%, or 60% of the culture flask volume was kept in contact with the cell culture in liquid medium and agitated at 100 rpm. In the growth conditions and cultivation system used in this study (see Materials and Methods), the optimal growth rate for *H. mediterranei* was observed in the culture with 60% air phase in the flask, thus emphasizing the importance of oxygen for this haloarchaeon (Figure 1A). Experiments with 80% air phase in the flask did not suppose a significant improvement of the harchaeal productivity (data not shown).

The effect of culture agitation speed on the growth rate of *H. mediterranei* was also evaluated using three different conditions: no agitation (0 rpm), 100 rpm, or 150 rpm, at 60% air phase. Figure 1B shows that an optimal growth rate of the haloarchaeon was obtained in cultures agitated at 150 rpm, but significant growth was also observed at 100 rpm. No growth was observed in non-gitated cultures (0 rpm), thus confirming that good aeration is absolutely essential to sustain the haloarchaeal growth. These findings are consistent with those reported for *H. alexandrinus*, the optimal growth of which was obtained in 100 mL culture medium in 500 mL flasks (80% air phase) [16], and *Halorubrum* sp. SHI, which required agitation at 550 rpm for optimal growth in 50 mL of culture medium in 250 mL flasks (80% air phase) [17].

For the operation, we used conditions of agitation speed of 150 rpm and 60% air phase in the culture flasks. Under these conditions, *H. mediterranei* showed a generation time of 33.6 h and maximum productivity of 22.16 g dry weight/L. This yield is significantly better than that obtained previously, not only with *H. mediterranei* [13], but also other aerobic haloarchaea [14]. Apart from the availability of oxygen, growth of *H. mediterranei* also depends on salinity, pH, and temperature of the culture medium [18]. According to Schneegurt (2012) [19], a 10% increase in salinity reduces oxygen solubility in the culture by approximately 50%, which might have an impact on the availability of oxygen for the haloarchaea cells. Oxygen solubility also decreases as the temperature increases or pH decreases, thus indicating that salinity, temperature, and pH have a complex influence on the growth rate of *H. mediterranei* cultures. 

Light is usually important for the regulation of carotenoid synthesis in many types of microorganisms. However, we found no differences either in pigmentation or in biomass concentration of *H. mediterranei* when cultivated in absence or presence of light (data not shown). The effect of light on pigmentation of halophilic archaea greatly depends on species and strains. For instance, strains *Hbt. salinarum* ATCC 33170, *Hbt. salinarum* ATCC 43214 and *Hfx. alexandrinus* TM (JCM 10717T), showed no difference in pigmentation when cultivated in the absence or in the presence of light. However, the pigment composition of *Hbt. salinarum* JCM 10927 alters according to light conditions, particularly by increasing the bacterioruberin content and decreasing the content of C40 carotenoids [7]. Thus, the effect of light should be studied for each specific halophilic microorganism.

### 2.2. Use of RSM to Optimize Culture Conditions for Growth and Carotenoid Production by H. mediterranei

**Figure 2:**
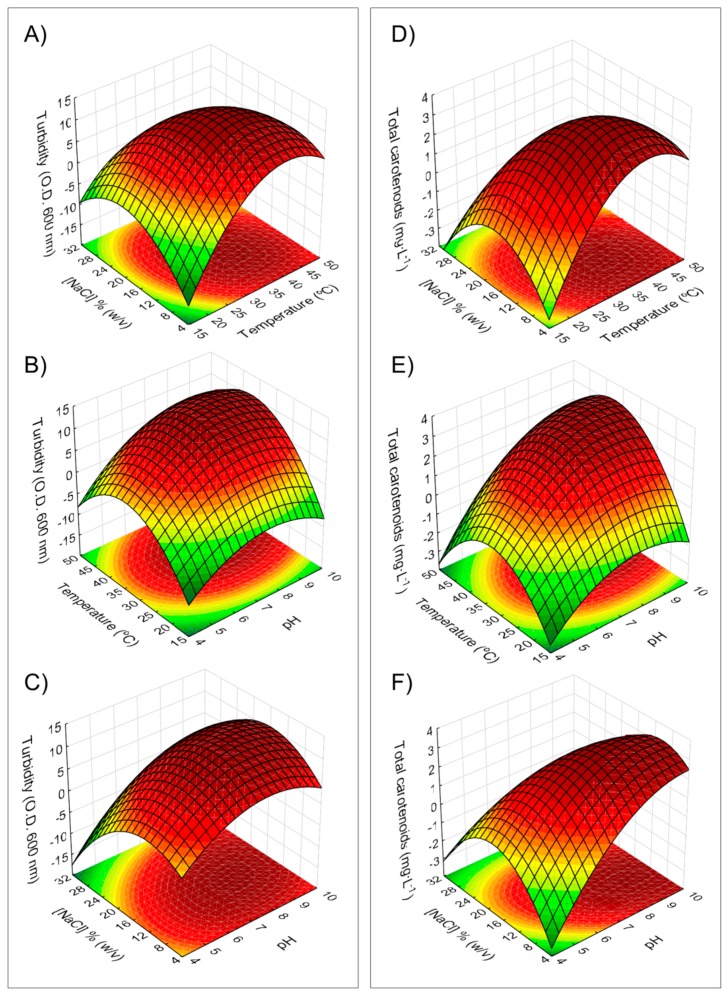
The 3-D-surface and contour response plots generated from a quadratic model representing the combined effects of temperature, pH, and salinity on the growth rate (**A**–**C**) and carotenoids content (**D**–**F**) by liquid cultures of *Hfx. mediterranei*. The interactions between salinity and temperature (**A**) and (**D**); pH and temperature (**B**) and (**E**), and pH and salinity (**C**) and (**F**) were analyzed. Other details of experimental conditions are stated in the Materials and Methods section.

Central composite design (CCD) was used to define the experimental growth conditions, which should obtain the predictive model for optimal growth and carotenoids production by *H. mediterranei*. As can be seen in Figure 1B, the culture at 60% air phase and an agitation rate of 150 rpm is at the late logarithmic phase on day 4, which seems adequate for measurements in the CCD experiments. Accordingly, 20 cultivation experiments were run on an orbital shaker using the parameters of agitation speed and air volume fraction defined above (150 rpm and 60%, respectively). The CCD and yield parameters are summarized in Table 1, which determined the response models in three-dimensional surfaces for the variables of haloarchaea growth (Figure 2A–C) and total carotenoid content (Figure 2D–F). According to the model, optimal growth of *H. mediterranei* should be obtained at 36.51 °C, pH of 8.20, and 15.01% (*w*/*v*) NaCl. 

The following equation could be used to predict the O.D. at 600 nm under different conditions:

(1) $$\begin{array}{ll} O.D.600\;nm= & -85.1+8.74\cdot X_{2}+2.681\cdot X_{1}+1.729\cdot X_{3}-0.681\cdot X_{2}^{2\;}-0.03635\cdot X_{1}^{2}\\ & -0.04710\;X_{3\;}^{2\;}+0.0394\;X_{2}\cdot X_{1\;}+\;0.0639\;X_{2}\cdot X_{3}-\;0.02318\;X_{1}\cdot X_{3}\; \end{array}$$

where *X*_1_, *X*_2_, *X*_3_ denote temperature, pH, and salinity, respectively (see Table 3).

On the other hand, the maximum total carotenoid content in cultures of *H. mediterranei* cells should be observed at 36.81 °C, pH of 8.96, and 12.03% of NaCl. The carotenoid content at any point during the culture and different conditions could be predicted according to the following equation:

(2) $$\;Carotenoids\left(\frac{mg}{L}\right)=\;-27.78+2.913\cdot X_{2}+\;0.647\cdot X_{1\;}+\;1.027\cdot X_{3\;}\;\;-0.1692\cdot X_{2}^{2}-\;0.00974\cdot X_{1}^{2}-0.01612\cdot X_{3\;}^{2}+\;0.0171\;X_{2}\cdot X_{1}-\;0.0419\;X_{2}-X_{3}-0.00718\;X_{1}-X_{3}\;$$

Using the one-factor-at-a-time approach, optimal conditions to produce carotenoids (2.06 mg/g dry weight of cells) by *H. alexandrinus* were 37 °C, pH of 7.2, and 25% NaCl [16], which are considerably different to this study, thus reiterating the importance of the haloarchaea species used and the interactions between factors.

### 2.3. Validation of the Optimal Conditions for Growth and Total Carotenoid Production by H. mediterranei

**Figure 3:**
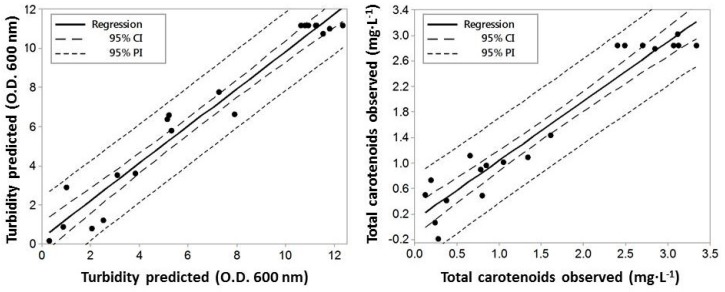
Theoretical values of response variables predicted from the respective models and observed values of the experimental design with a *p*-value < 0.05 for the growth rate and total intracellular carotenoids by *Hfx. mediterranei*. The growth and carotenoids content are as described in Materials and Methods. CI = reliable interval and PI = predicted interval.

The accuracy of the model was verified by analyzing the predicted and observed experimental results. Three experiments were carried out to determine the reliability of optimal conditions predicted by the models of Equations (1) and (2), using the data obtained for biomass and total carotenoid content, respectively. Figure 3 shows that high values of R^2^ (93.1%) and adjusted R^2^ (92.7%) highlight the agreement between predicted and observed experimental values. Hence, an acceptable relationship between independent variables (temperature, pH, and salinity) and response variables (growth and total carotenoids) was proved. The highest biomass production (21.95 ± 1.57 g dry weight/L) and total carotenoid content (3.74 ± 0.20 mg/L) were very close to the values estimated using RSM at the optimal conditions (20.18 ± 1.02 g dry weight/L, and 3.34 ± 0.29 mg/L, respectively) indicating that RSM is effective in fixing culture conditions where several variables could influence the final result. It can also predict results for other potential culture conditions of the haloarchaea, as reported by the authors of Reference [14], in similar studies on *Halorubrum* sp. TZB126.

The data demonstrates for the first time in *H. mediterranei* that the RSM approach might be used to predict optimal conditions for large scale carotenoid production. 

### 2.4. Carotenoid Profile Obtained from H. mediterranei

**Figure 4:**
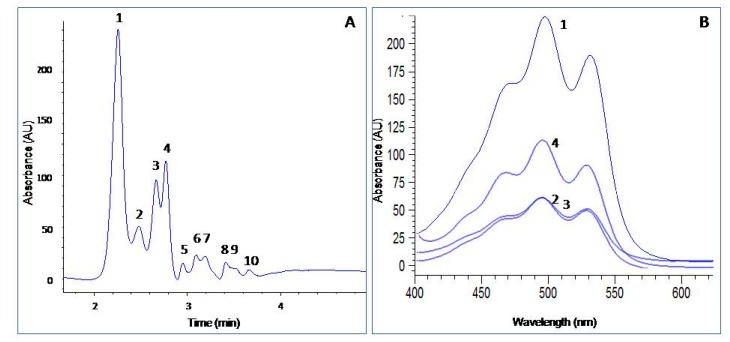
HPLC analysis of the carotenoids present in *Hfx. mediterranei* (**A**) and the absorption spectrum of isolated bacterioruberin (**B**). Peaks 1–4 are the isomers of bacterioruberin, peaks 5, 6, 7, 8, 9, and 10 are C_50_ carotenoid derivatives from bacterioruberin. Further experimental conditions are indicated in the Materials and Methods.

Bacterioruberin is the major C_50_ carotenoid in all the archaeal strains, however, β-carotene, lycopene, astaxanthin, and canthaxanthin were also found in these organisms [3,20]. The profile of the carotenoid obtained from *H. mediterranei* was analyzed using HPLC, and the results revealed a chromatogram with 10 peaks where bacterioruberin (89.13%) was the major compound produced under the optimal conditions used for carotenoid production (Figure 4A). Peaks 1–4 had the same molecular weight (Table 2). Figure 4B shows a similar 3-finger type absorption spectrum for these carotenoid fractions with typical bacterioruberin absorption maxima at 468, 495, and 530 nm, thereby indicating that they were isomers of the main carotenoid, probably 13-cis-bacterioruberin and 9-cis-bacterioruberin, respectively [9]. However, the definitive structures of peaks 1-4 cannot be elucidated until further studies are done.

Figure 4A also reveals other minor peaks corresponding to chemically modified bacterioruberin-derived compounds such as monoanhydrobacterioruberin and bisanhydrobacterioruberin [13], the molecular weights differed from bacterioruberin (Table 2). The other peaks observed in the chromatogram corresponded to unknown carotenoids. Calo et al. (1995) [21] reported 3-hydroxyechinenone as a major carotenoid of *H. mediterranei*. However, the existence of 3-hydroxyechinenone in *H. mediterranei* has not been referred to in any other paper after 1995. Other studies on *H. mediterranei* reported 70% and 52.4% bacterioruberin in the carotenoid fraction [8,13], respectively, indicating the influence of the culture conditions on the yields of carotenoids and composition of the haloarchaea. By increasing the amount of magnesium sulfate in the medium, the relative ratio of bacterioruberin was increased, reaching a constant level at 8% (*w*/*v*) of magnesium [13]. In our study, 2% magnesium sulfate was used in the culture medium for *H. mediterranei*. The bacterioruberin content obtained from other haloarchaea is highly variable, as shown in Reference [14], (98.1% in *Halorubrum* sp.); [10], (68.1% in *Haloarcula japonica*); and [22], (49.2% in *Halobacterium* SP–2 and 55.3% in *Halorubrum* SP–4). These data show that *H. mediterranei* grown under the conditions stated in this work contains high levels of bacterioruberin compared with other haloarchaea.

### 2.5. Bacterioruberin Production by H. mediterranei

The maximum carotenoid yield in our experiments was 3.74 mg/L (equivalent to 23.51 mg/g dry weight), which is different from that reported in other haloarchaea. The yield of carotenoids in haloarchaea mainly depended on the strain and on the culture conditions used. *H. alexandrinus* accumulates 2.6 mg/g dry weight [16]; *Halobacterium salinarum*, 45 µg/g dry weight and *Halococcus morrhuae*, 89 µg/g dry weight [9], *Halobacterium halobium*, 7.63 mg/L [23], *Halorubrum* sp., 10.78 mg/L [14], *H. mediterranei*, 125 mg/L [8], *Haloarcula japonica*, 335 µg/g dry weight [10]; *Halorubrum* sp. SH1, 25 mg/L [17], and *Haloterrigena turkmenica*, 32 µg/g dry weight [24]. However, in most cases, the corresponding information concerning biomass production and/or cell viability, under the conditions used for carotenoid production are absent, which makes it difficult to select one strain of haloarchaea for large-scale production of carotenoids.

The chosen strategy significantly affects the final costs, as the following options indicate: (i) one-step production under optimal growth conditions, in which the carotenoid production is directly linked to the biomass production of the cultures, or (ii) a two-step system, whereby the first step of biomass production under optimal growth conditions is followed by the second phase of cultivation under stress to promote biosynthesis and accumulation of carotenoids. In our study, the salt content in the culture medium seemed to establish the best conditions for carotenoid production. According to literature, haloarchaea require high salt concentration for optimal growth, while maximum carotenoid production is achieved when cells are under stress produced by low external salinity. Chen et al. (2015) [8] showed that *H. mediterranei* growing at 40 S/m conductivity (a measurement of salt concentration) in saline medium accumulated 125 mg/L of total carotenoids; however, if the conductivity of the medium was decreased to 25 S/m, the pigments could be increased to a maximum value of 555.6 mg/L. From Equations (2) and (3), we estimated that *H. mediterranei* can produce 3.34 mg/L of carotenoids, while the theoretical value for the growth of haloarchaea under such conditions is 18.51 g dry weight/L, which corresponds to a loss of about 7.5% of the biomass productivity. Thus, the option of a one-step process is adequate for the high-scale bacterioruberin production by *H. mediterranei*. Fixing the optimal conditions for carotenoid production increases the biotechnological value of this halophilic microorganism. 

Calegari-Santos et al. (2016) [7] reviewed the effect of different stress conditions on carotenoids production in halophilic archaea. In addition to the variables considered in this work, the C-source and the presence or absence of metals is also relevant. However, the effect of *N*-starvation and other nutritional stress factors remain to be examined. 

## 3. Materials and Methods

### 3.1. Microorganism

The highly halophilic archaeon *Haloferax mediterranei*, strain R4 (ATCC 33500T), used in this study, was provided by Dr. Rosa María Martínez from the Department of Agrochemistry and Biochemistry, University of Alicante, Spain. This archaeon was first isolated and reported by the authors of Reference [25], from saline water at Santa Pola in Alicante (Spain).

### 3.2. Growth Conditions and Biomass Quantification

The haloarchaea were grown in a basal culture medium as formulated in Reference [13], containing (per liter): Glucose, 10 g; NaCl, 156 g; MgCl_2_·6H_2_O, 13 g; MgSO_4_·7H_2_O, 20 g; CaCl_2_·6H_2_O, 1 g; KCl, 4 g; NaHCO_3_, 0.2 g; NaBr, 0.5 g; yeast extract, 5 g; and the pH was adjusted to 7.0 by addition of diluted KOH or HCl. The mother culture was prepared in 100 mL of liquid medium contained in a 250 mL flask and incubated at 37 °C and 150 rpm on an orbital shaker until the exponential phase of growth was achieved (standard conditions). This culture was used as inoculum at 10% (*v*/*v*), in all the experiments. The growth was determined by measuring the turbidity of the culture at 600 nm using a UV-Vis spectrophotometer (Thermo Spectronic, Genesis, Waltham, MA, USA). The dry weight was determined using 1 mL sample of the corresponding culture, which was filtered through a pre-weighed membrane (ϕ = 0.2 μm) and the retained cells were washed on the filter using 5 mL of 1% NaCl (*w*/*v*) solution. The membrane was then dried at 80 °C until a constant weight was reached. A control with 1 mL of uninoculated culture medium was run in parallel. The weight was later deducted from the sample. Culture with OD of 1.0 at 600 nm had a dry weight of 1.60 g/L.

### 3.3. Extraction, Quantification, and Analysis of Pigments

For extraction of carotenoids, the culture samples (10 mL) were centrifuged at 3500× *g* for 45 min, the harvested cells were lysed by freeze/thawing, and finally, the biological material was resuspended in 1 mL of pure acetone and kept overnight at 4 °C. The suspension was centrifuged at 3500× *g* for 5 min. The total carotenoid content of the supernatant was determined by measuring the absorbance at 494 nm and calculated using an extinction coefficient, ε (1%), of 2540, according to the following expression: mg/L = (OD_494_/2540) × 10^4^.

The HPLC analysis of carotenoids in acetone was performed using a Poroshell 120-C18 column (Agilent, Santa Clara, CA, USA) (3 × 50 mm, 2.7 μm) on an Agilent 1200 series system (Santa Clara, CA, USA) equipped with a diode array detector scanning from 400 to 690 nm. To determine the mass spectra of the different compounds, a 6410 Triple Quad LC/MS system (Agilent, Santa Clara, CA, USA) was used equipped with an electrospray ionization source (ESI) operating in positive scan mode (m/z range of 300–900), with ±0.1 u.m.a. precision, and controlled by Mass Hunter Workstation Software (Agilent, B.05.00, Santa Clara, CA, USA). The following specific working conditions were used: capillary voltage 4000 V, gas flow rate 10 L m^−1^, gas temperature 300 °C, and nebulizer pressure 35 psi [17].

### 3.4. Response Surface Methodology Experimental Design

The one-factor-at-a-time approach used to analyze a problem based on three or more parameters overlooks the interactions between different factors [26]. To address these issues, RSM was used to identify the optimal value to be applied in order to determine the main effect as well as any significant interactions between factors that may exert important effects on response variables [14,27,28]. A central and axial points design (CCD) approach was used to optimize the culture conditions for both cell growth (O.D. at 600 nm) and total carotenoid content (mg/L) by *H. mediterranei*. In this study, temperature, salinity, and pH were considered for the CCD analysis. They were investigated at five different levels within the following ranges: temperature (15–50 °C), pH (4−10), and NaCl concentration (5–28%, *w*/*v*) in order to deduce the optimum values of growth and carotenoid content. The code and actual values of the variables are presented in Table 3.

A 2^3^ full-factorial experimental design with six-axial points and six central points was chosen [29]. The relationship between the response variable and the independent variables was fitted by a predictive quadratic polynomial equation. The quality of fit for the second-order model equation was expressed by the coefficient of determination (R^2^) and its statistical significance was determined using the *p*-value. To provide an adequate degree of freedom (df = 5) for estimation of pure error, calculations at the central point were repeated six times. The regression equation used is described as follows:

(3) $$\;y\;=\;\beta_{0}+\;\overset{3}{\underset{i=1}{\sum }}\beta_{i}x_{i}+\;\overset{3}{\underset{i=1}{\sum }}\beta_{ii}x_{i}^{2}+\;\overset{3}{\underset{i,j=1}{\sum }}\beta_{ij}x_{i}x_{j}\;$$

where y represents the predicted response variables (growth or total carotenoid); $\beta_{0}$ is a constant, $\beta_{i}$ is the linear coefficient, $\beta_{ii}$ is the quadratic coefficient, $\beta_{ij}$ is the interaction coefficient of the model, respectively, and $x_{i}$ and $x_{j}$ (*i* = 1, 3; *j* = 1, 3; *i* ≠ *j*) represent the non-coded independent variables (temperature, pH, and salinity).

### 3.5. Statistical Analysis

The data analysis for model construction was performed using Minitab 17.1.0.0 software (Minitab Inc., State College, PA, USA), based on the response surface methodology. The model was statistically tested using analysis of variance (ANOVA) to test the significance and adequacy of the model. Regression analysis was used to obtain the coefficients of a second order polynomial. Data are presented as the average of three independent experiments. Statistical significance was determined by *p*-value at *p* < 0.05. The three-dimensional surface plot and contour plot performed by the regression model were drawn using the Statistica software package (version 11.0, StatSoft, Palo Alto, CA, USA) to highlight the effects on the independent variables and corresponding effects on the response variables.

## 4. Conclusions

Temperature (36.51 °C or 36.81 °C), pH (8.20 or 8.96), and salinity (15.01 or 12.03%, *w*/*v*) are the optimal conditions for the *H. mediterranei* biomass and carotenoid production. Bacterioruberin, a carotenoid of high antioxidant capacity, is the major C_50_ carotenoid of *H. mediterranei*. RMS approach serves to accurately predict both the biomass and carotenoid production by the haloarchaeon at any temperature, pH, and salinity of the media, which is valuable for performing C_50_ carotenoid production—particularly bacterioruberin—by *H. mediterranei* in a one-step process.

## Figures and Tables

**Table 1:** Central composite design (CCD) matrix and the responses of growth and total carotenoid content at different temperature, pH, and salinity levels. Std Order: Standard Order.

	Independent Variables						Responses	
	Coded Levels							
Std Order	Temperature (°C)		pH		Salinity (NaCl% *w*/*v*)		Turbidity (O.D.600 nm)	Total Carotenoids (mg/L)
1	−1	23.8	−1	5.5	−1	9.8	2.53	0.28
2	1	23.8	−1	8.5	−1	9.8	3.09	1.61
3	−1	41.3	1	5.5	−1	9.8	7.88	0.80
4	1	41.3	1	8.5	−1	9.8	11.79	3.12
5	−1	23.8	−1	5.5	1	23.3	0.88	1.34
6	1	23.8	−1	8.5	1	23.3	5.31	1.05
7	−1	41.3	1	5.5	1	23.3	2.04	0.24
8	1	41.3	1	8.5	1	23.3	7.25	0.78
9	−1.68	32.5	0	4.5	0	16.5	1.01	0.19
10	1.68	32.5	0	9.5	0	16.5	11.51	2.85
11	0	17.8	1.68	7.0	0	16.5	0.29	0.13
12	0	47.2	1.68	7.0	0	16.5	5.14	0.85
13	0	32.5	0	7.0	−1.68	5.1	5.22	0.66
14	0	32.5	0	7.0	1.68	27.9	3.82	0.37
15 *	0	32.5	0	7.0	0	16.5	10.93	3.34
16 *	0	32.5	0	7.0	0	16.5	11.25	3.13
17 *	0	32.5	0	7.0	0	16.5	10.78	2.40
18 *	0	32.5	0	7.0	0	16.5	10.62	2.71
19 *	0	32.5	0	7.0	0	16.5	11.21	2.50
20 *	0	32.5	0	7.0	0	16.5	12.34	3.07

* Central point values contributing to the degree of freedom for pure error calculation.

**Table 2:** Tentative identification of carotenoids present in *Haloferax mediterranei*. BR: bacterioruberin; MABR: monoanhydrobacterioruberin; BABR: bisanhydrobacterioruberin.

Peak	Carotenoid	Retention Time (min)	λmax (nm)	Molecular Ion (*m*/*z*) M^+^	Fragments Profile
1	BR	2.325	468, 496, 530	740.7	723.7, 705.7, 687.7, 666.7, 561.5, 515,1
2	BR	2.553	468, 494, 528	740.7	723.7, 705.7, 681,6, 666.8, 655.6, 627.6
3	BR	2.740	468, 496, 528	740.7	723.7, 705.7, 682.6, 669.6, 665.6
4	BR	2.816	464, 494, 524	740.7	723.7, 705.7, 682.6, 665.6
5	MABR	3.021	470, 500, 534	737.7	725.6, 709.6, 699.7
6	BABR	3.168	460, 488, 520	705.7	681.6, 669.7, 579.7, 522.7
7	BABR	3.233	456, 485, 526	705.7	699.7, 671.7, 668.7, 647.6, 579.6
8	BABR	3.508	472, 498, 532	705.7	699.7, 687.7, 671.7, 653.8, 607.6
9	BABR	3.620	468, 490, 520	705.7	699.7, 671.7, 653.8, 550.6

**Table 3:** The coded and actual values of experimental variables used in the central composite design (CCD).

Independent Variables	Symbols	Levels				
		−1.68 *	−1	0	1	1.68 *
Temperature (°C)	*X*_1_	17.8	23.8	32.5	41.3	47.2
pH	*X*_2_	4.5	5.5	7.0	8.5	9.5
[NaCl]% (*w*/*v*)	*X*_3_	5.15	9.75	16.50	23.25	27.85

* Alpha values used for axial points in this study.
